# 9-(3-Fluoro­phen­yl)-3,3,6,6-tetra­methyl-1,2,3,4,5,6,7,8,9,10-deca­hydro­acridine-1,8-dione

**DOI:** 10.1107/S1600536812050556

**Published:** 2012-12-15

**Authors:** Rajni Kant, Vivek K. Gupta, Kamini Kapoor, D. R. Patil, S. D. Jagadale, Madhukar B. Deshmukh

**Affiliations:** a X-ray Crystallography Laboratory, Post-Graduate Department of Physics & Electronics, University of Jammu, Jammu Tawi 180 006, India; b Department of Chemistry, Shivaji University, Kolhapur, 416 004 (MS), India

## Abstract

In the title mol­ecule, C_23_H_26_FNO_2_, the central ring of the acridinedione system adopts a slight boat conformation and the four essentially planar atoms of this ring [maximum deviation = 0.019 (1) Å] form a dihedral angle of 89.98 (6)° with the benzene ring. The two outer rings of the acridinedione system adopt sofa conformations. In the crystal, N—H⋯O hydrogen bonds link the mol­ecules, forming chains along [001].

## Related literature

For applications of acridines, see: Murugan *et al.* (1998 ▶); Leon *et al.* (2008 ▶). Josephrajan *et al.* (2005 ▶); Srividya *et al.* (1998 ▶, 1996 ▶). For related structures, see: Balamurugan *et al.* (2009 ▶); Zhao & Teng (2008 ▶); Kant *et al.* (2013 ▶). For ring conformations, see: Duax & Norton (1975 ▶).
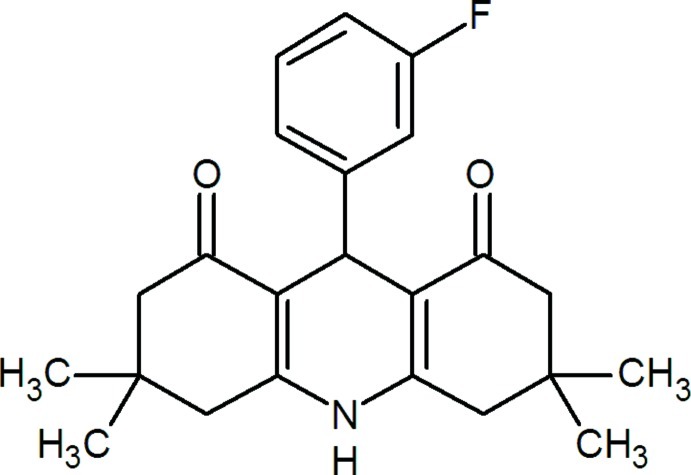


## Experimental

### 

#### Crystal data

C_23_H_26_FNO_2_
*M* *_r_* = 367.45
Monoclinic, 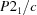

*a* = 11.0505 (3) Å
*b* = 12.8264 (3) Å
*c* = 13.8548 (3) Å
β = 100.215 (2)°
*V* = 1932.63 (8) Å^3^
*Z* = 4
Mo *K*α radiation
μ = 0.09 mm^−1^
*T* = 293 K
0.3 × 0.2 × 0.2 mm

#### Data collection

Oxford Diffraction Xcalibur Sapphire3 diffractometer
Absorption correction: multi-scan (*CrysAlis PRO*; Oxford Diffraction, 2010 ▶) *T*_min_ = 0.897, *T*_max_ = 1.000
30330 measured reflections
3789 independent reflections
2922 reflections with *I* > 2σ(*I*)
*R*_int_ = 0.041

#### Refinement

*R*[*F* ^2^ > 2σ(*F* ^2^)] = 0.044
*wR*(*F* ^2^) = 0.118
*S* = 1.03
3789 reflections
248 parameters
H-atom parameters constrained
Δρ_max_ = 0.21 e Å^−3^
Δρ_min_ = −0.30 e Å^−3^

### 

Data collection: *CrysAlis PRO* (Oxford Diffraction, 2010 ▶); cell refinement: *CrysAlis PRO*; data reduction: *CrysAlis PRO*; program(s) used to solve structure: *SHELXS97* (Sheldrick, 2008 ▶); program(s) used to refine structure: *SHELXL97* (Sheldrick, 2008 ▶); molecular graphics: *ORTEP-3* (Farrugia, 2012 ▶); software used to prepare material for publication: *PLATON* (Spek, 2009 ▶).

## Supplementary Material

Crystal structure: contains datablock(s) I, global. DOI: 10.1107/S1600536812050556/lh5569sup1.cif

Structure factors: contains datablock(s) I. DOI: 10.1107/S1600536812050556/lh5569Isup2.hkl

Supplementary material file. DOI: 10.1107/S1600536812050556/lh5569Isup3.cml

Additional supplementary materials:  crystallographic information; 3D view; checkCIF report

## Figures and Tables

**Table 1:** Hydrogen-bond geometry (Å, °)

*D*—H⋯*A*	*D*—H	H⋯*A*	*D*⋯*A*	*D*—H⋯*A*
N10—H10⋯O1^i^	0.86	2.14	2.990 (2)	168

Symmetry code: (i) 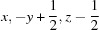.

**Fig. 1.**
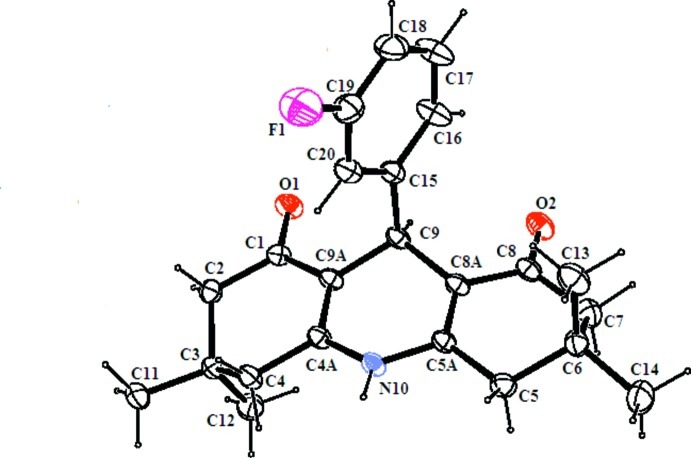
The molecular structure of the title compound with ellipsoids drawn at the 40% probability level. H atoms are shown as small spheres of arbitrary radii.

**Fig. 2.**
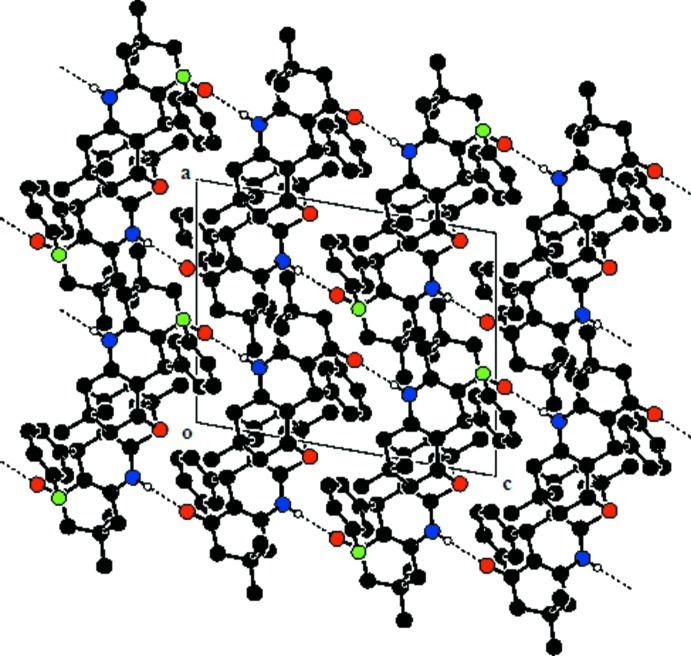
The packing arrangement of molecules viewed along the b axis. The dotted lines show intermolecular N—H···O hydrogen bonds.

## supplementary crystallographic information

### Comment

The 1,4- dihydropyridine (DHP) nucleus act as a versatile intermediate for the synthesis of several pharmaceuticals together with those of cardiovascular drugs and as a calcium channel modulators, laser dyes and photo initiators (Leon *et al.*, 2008). Acridines, the earliest known antibiotics, are toxic towards bacteria. Some acridinedione derivatives show good inhibition against the pathogen Vibrio isolate-I (Josephrajan *et al.*, 2005). Certain acridine-1,8-diones exhibit fluorescence activities (Murugan *et al.*, 1998) and a few acridinedione derivatives also show photophysical (Srividya *et al.*, 1998) and electrochemical properties (Srividya *et al.*, 1996). Thus, the accurate description of crystal structures of substituted acridinediones are expected to provide useful information on the role of substituents in influencing molecular conformation which has a direct relationship to biological activity. This paper deals with the crystal structure of a 3-fluorophenyl substituted tetramethyl acridinedione, (I).

In (I) (Fig.1), all bond lengths and angles are normal and correspond to those observed in related structures (Balamurugan *et al.*,2009; Zhao & Teng 2008; Kant *et al.*, 2013). The central ring (C4A/C5A/C8A/C9A/C9/N10) of the acridinedione moiety adopts a boat conformation (ΔCs(C9) = 4.85 & ΔCs (C9A—C4A) = 14.52) and the four essentially planar atoms (C4A/C5A/C8A/C9A) of this ring (maximum deviation = 0.019 (1) Å) form a dihedral angle of 89.98 (6)° with benzene ring. Both the outer rings adopt sofa conformations (ΔCs (C3) = 1.01; ΔCs (C8A) = 6.56) (Duax & Norton, 1975). In the crystal, N10—H10···O1^i^ hydrogen bonds (Table 1) link molecules to form link molecules to form one-dimensional chains along [001] (Fig. 2).

### Experimental

In a 50 ml rounded bottom flask, a mixture of dimedone (2 mmole), 3-fluoro benzaldehyde (1 mmole) and ammonium acetate (1.2 mmole) in mixture of aq. ethanol (7 ml) was stirred at RT for 5 min. To this[CMIM][HSO4](3-carboxy methyl-1-methylimidazolium bisulfate) (20 mol %) was added and the reaction mixture heated at 348-353K for 1.5 hrs. The progress of the reaction was monitored by TLC. After completion of the reaction, the mixture was gradually cooled to RT and poured on ice water under stirring. The precipitate was dried. Diffraction quality single crystals were grown from a solution of the title compound in ethanol.

M.P.: 573 K, Yield: 87%. IR(KBr): 3217, 3070, 2954, 1627 cm-1. 1H NMR (300 MHz, DMSO-d6): δ = 9.1 (brs, 1H,NH); 7.6–7.2 (m, 4H,Ar—H); 5.5 (s, 1H,CH); 3.3–2.5 (m,8*H*,CH2); 1.6 (s,6*H*, CH3); 1.4 (s, 6H, CH3).

### Refinement

All H atoms were positioned geometrically and were treated as riding on their parent C atoms, with N—H distance of 0.86 Å, C—H distances of 0.93–0.98 Å and with *U*_iso_(H) = 1.2*U*_eq_(C/N) or 1.5*U*_eq_(methyl C).

### Crystal data

**Table:**

C_23_H_26_FNO_2_	*F*(000) = 784
*M**_r_* = 367.45	*D*_x_ = 1.263 Mg m^−^^3^
Monoclinic, *P*2_1_/*c*	Mo *K*α radiation, λ = 0.71073 Å
Hall symbol: -P 2ybc	Cell parameters from 11314 reflections
*a* = 11.0505 (3) Å	θ = 3.5–29.1°
*b* = 12.8264 (3) Å	µ = 0.09 mm^−^^1^
*c* = 13.8548 (3) Å	*T* = 293 K
β = 100.215 (2)°	Block, yellow
*V* = 1932.63 (8) Å^3^	0.3 × 0.2 × 0.2 mm
*Z* = 4	

### Data collection

**Table:**

Oxford Diffraction Xcalibur Sapphire3 diffractometer	3789 independent reflections
Radiation source: fine-focus sealed tube	2922 reflections with *I* > 2σ(*I*)
Graphite monochromator	*R*_int_ = 0.041
Detector resolution: 16.1049 pixels mm^-1^	θ_max_ = 26.0°, θ_min_ = 3.5°
ω scans	*h* = −13→13
Absorption correction: multi-scan (*CrysAlis PRO*; Oxford Diffraction, 2010)	*k* = −15→15
*T*_min_ = 0.897, *T*_max_ = 1.000	*l* = −17→17
30330 measured reflections	

### Refinement

**Table:**

Refinement on *F*^2^	Primary atom site location: structure-invariant direct methods
Least-squares matrix: full	Secondary atom site location: difference Fourier map
*R*[*F*^2^ > 2σ(*F*^2^)] = 0.044	Hydrogen site location: inferred from neighbouring sites
*wR*(*F*^2^) = 0.118	H-atom parameters constrained
*S* = 1.03	*w* = 1/[σ^2^(*F*_o_^2^) + (0.0534*P*)^2^ + 0.5565*P*] where *P* = (*F*_o_^2^ + 2*F*_c_^2^)/3
3789 reflections	(Δ/σ)_max_ = 0.002
248 parameters	Δρ_max_ = 0.21 e Å^−^^3^
0 restraints	Δρ_min_ = −0.30 e Å^−^^3^

### Special details

**Table:**

Experimental. *CrysAlis PRO*, Oxford Diffraction Ltd., Version 1.171.34.40 (release 27–08-2010 CrysAlis171. NET) (compiled Aug 27 2010,11:50:40) Empirical absorption correction using spherical harmonics, implemented in SCALE3 ABSPACK scaling algorithm.
Geometry. All e.s.d.'s (except the e.s.d. in the dihedral angle between two l.s. planes) are estimated using the full covariance matrix. The cell e.s.d.'s are taken into account individually in the estimation of e.s.d.'s in distances, angles and torsion angles; correlations between e.s.d.'s in cell parameters are only used when they are defined by crystal symmetry. An approximate (isotropic) treatment of cell e.s.d.'s is used for estimating e.s.d.'s involving l.s. planes.
Refinement. Refinement of *F*^2^ against ALL reflections. The weighted *R*-factor *wR* and goodness of fit *S* are based on *F*^2^, conventional *R*-factors *R* are based on *F*, with *F* set to zero for negative *F*^2^. The threshold expression of *F*^2^ > σ(*F*^2^) is used only for calculating *R*-factors(gt) *etc*. and is not relevant to the choice of reflections for refinement. *R*-factors based on *F*^2^ are statistically about twice as large as those based on *F*, and *R*- factors based on ALL data will be even larger.

### Fractional atomic coordinates and isotropic or equivalent isotropic displacement parameters (Å^2^)

**Table:**

	*x*	*y*	*z*	*U*_iso_*/*U*_eq_	
F1	0.41296 (13)	0.61151 (11)	0.95736 (11)	0.0872 (5)	
O1	0.37340 (11)	0.15628 (10)	1.02926 (8)	0.0458 (3)	
O2	−0.05752 (11)	0.26403 (9)	0.87999 (9)	0.0447 (3)	
C1	0.40151 (14)	0.17311 (12)	0.94855 (10)	0.0314 (3)	
C2	0.52391 (14)	0.13764 (13)	0.92694 (11)	0.0350 (4)	
H2A	0.5477	0.0743	0.9637	0.042*	
H2B	0.5849	0.1905	0.9503	0.042*	
C3	0.52646 (14)	0.11663 (12)	0.81853 (11)	0.0326 (4)	
C4	0.47300 (15)	0.21159 (14)	0.75919 (11)	0.0369 (4)	
H4A	0.5312	0.2687	0.7715	0.044*	
H4B	0.4616	0.1949	0.6899	0.044*	
C4A	0.35251 (14)	0.24594 (11)	0.78386 (11)	0.0299 (3)	
C5	0.06856 (15)	0.34840 (14)	0.62736 (11)	0.0374 (4)	
H5A	0.0487	0.2898	0.5831	0.045*	
H5B	0.1117	0.3997	0.5947	0.045*	
C5A	0.15152 (14)	0.31198 (12)	0.71821 (11)	0.0300 (3)	
C6	−0.05098 (15)	0.39660 (14)	0.64830 (12)	0.0382 (4)	
C7	−0.10509 (15)	0.32075 (15)	0.71400 (13)	0.0436 (4)	
H7A	−0.1781	0.3519	0.7317	0.052*	
H7B	−0.1303	0.2579	0.6769	0.052*	
C8	−0.01831 (15)	0.29119 (12)	0.80649 (12)	0.0333 (4)	
C8A	0.11243 (14)	0.29285 (11)	0.80350 (11)	0.0299 (3)	
C9	0.20404 (14)	0.27571 (12)	0.89720 (11)	0.0311 (3)	
H9	0.1689	0.2262	0.9385	0.037*	
C9A	0.32076 (14)	0.23003 (12)	0.87301 (11)	0.0304 (3)	
N10	0.27307 (11)	0.29683 (10)	0.71127 (9)	0.0329 (3)	
H10	0.2999	0.3196	0.6606	0.039*	
C11	0.65972 (16)	0.09985 (16)	0.80536 (13)	0.0476 (5)	
H11A	0.6623	0.0897	0.7371	0.071*	
H11B	0.6926	0.0395	0.8418	0.071*	
H11C	0.7079	0.1599	0.8290	0.071*	
C12	0.45147 (17)	0.01935 (14)	0.78447 (14)	0.0487 (5)	
H12A	0.3677	0.0297	0.7923	0.073*	
H12B	0.4853	−0.0394	0.8230	0.073*	
H12C	0.4543	0.0065	0.7166	0.073*	
C13	−0.02574 (19)	0.50270 (14)	0.69808 (15)	0.0511 (5)	
H13A	−0.1012	0.5313	0.7116	0.077*	
H13B	0.0084	0.5490	0.6555	0.077*	
H13C	0.0315	0.4944	0.7584	0.077*	
C14	−0.14131 (19)	0.41061 (19)	0.55164 (15)	0.0616 (6)	
H14A	−0.1582	0.3441	0.5205	0.092*	
H14B	−0.1059	0.4562	0.5092	0.092*	
H14C	−0.2165	0.4404	0.5647	0.092*	
C15	0.22599 (15)	0.37969 (12)	0.95166 (11)	0.0332 (4)	
C16	0.1496 (2)	0.41131 (15)	1.01546 (14)	0.0534 (5)	
H16	0.0891	0.3662	1.0294	0.064*	
C17	0.1618 (2)	0.50844 (17)	1.05846 (16)	0.0634 (6)	
H17	0.1094	0.5278	1.1010	0.076*	
C18	0.2498 (2)	0.57702 (16)	1.03973 (14)	0.0558 (5)	
H18	0.2580	0.6429	1.0682	0.067*	
C19	0.32474 (18)	0.54449 (15)	0.97744 (14)	0.0503 (5)	
C20	0.31554 (16)	0.44858 (14)	0.93376 (12)	0.0437 (4)	
H20	0.3693	0.4298	0.8922	0.052*	

### Atomic displacement parameters (Å^2^)

**Table:**

	*U*^11^	*U*^22^	*U*^33^	*U*^12^	*U*^13^	*U*^23^
F1	0.0871 (10)	0.0698 (9)	0.1120 (11)	−0.0351 (8)	0.0372 (9)	−0.0326 (8)
O1	0.0544 (8)	0.0560 (8)	0.0294 (6)	0.0148 (6)	0.0140 (5)	0.0112 (5)
O2	0.0456 (7)	0.0442 (7)	0.0505 (7)	0.0011 (5)	0.0255 (6)	0.0061 (6)
C1	0.0385 (9)	0.0287 (8)	0.0280 (8)	0.0023 (6)	0.0083 (6)	0.0000 (6)
C2	0.0345 (9)	0.0362 (9)	0.0338 (8)	0.0049 (7)	0.0043 (7)	0.0029 (7)
C3	0.0305 (8)	0.0338 (9)	0.0339 (8)	0.0066 (7)	0.0069 (6)	−0.0026 (7)
C4	0.0370 (9)	0.0426 (10)	0.0343 (8)	0.0091 (7)	0.0154 (7)	0.0055 (7)
C4A	0.0342 (8)	0.0258 (8)	0.0315 (8)	0.0057 (6)	0.0110 (6)	0.0037 (6)
C5	0.0414 (9)	0.0411 (9)	0.0304 (8)	0.0066 (7)	0.0085 (7)	0.0025 (7)
C5A	0.0339 (8)	0.0260 (8)	0.0316 (8)	0.0054 (6)	0.0104 (6)	0.0010 (6)
C6	0.0336 (9)	0.0433 (10)	0.0376 (9)	0.0076 (7)	0.0064 (7)	0.0030 (7)
C7	0.0336 (9)	0.0462 (10)	0.0520 (10)	−0.0009 (8)	0.0100 (8)	−0.0008 (8)
C8	0.0381 (9)	0.0236 (8)	0.0412 (9)	0.0013 (6)	0.0149 (7)	−0.0021 (7)
C8A	0.0357 (8)	0.0242 (7)	0.0321 (8)	0.0051 (6)	0.0118 (6)	0.0021 (6)
C9	0.0367 (8)	0.0301 (8)	0.0299 (8)	0.0071 (6)	0.0153 (6)	0.0063 (6)
C9A	0.0366 (8)	0.0263 (8)	0.0304 (8)	0.0063 (6)	0.0118 (6)	0.0024 (6)
N10	0.0359 (7)	0.0377 (7)	0.0282 (6)	0.0085 (6)	0.0143 (5)	0.0087 (6)
C11	0.0371 (10)	0.0608 (12)	0.0459 (10)	0.0141 (8)	0.0097 (8)	−0.0022 (9)
C12	0.0481 (11)	0.0399 (10)	0.0565 (11)	0.0047 (8)	0.0044 (9)	−0.0111 (8)
C13	0.0585 (12)	0.0388 (10)	0.0592 (12)	0.0117 (9)	0.0189 (9)	0.0052 (9)
C14	0.0457 (11)	0.0861 (16)	0.0504 (11)	0.0153 (11)	0.0015 (9)	0.0114 (11)
C15	0.0390 (9)	0.0343 (9)	0.0273 (8)	0.0102 (7)	0.0087 (6)	0.0035 (6)
C16	0.0703 (13)	0.0418 (10)	0.0577 (12)	0.0033 (9)	0.0375 (10)	−0.0041 (9)
C17	0.0827 (16)	0.0515 (12)	0.0660 (13)	0.0086 (11)	0.0410 (12)	−0.0144 (10)
C18	0.0690 (13)	0.0433 (11)	0.0550 (12)	0.0076 (10)	0.0108 (10)	−0.0158 (9)
C19	0.0508 (11)	0.0471 (11)	0.0525 (11)	−0.0065 (9)	0.0080 (9)	−0.0068 (9)
C20	0.0439 (10)	0.0485 (11)	0.0414 (9)	0.0020 (8)	0.0145 (8)	−0.0071 (8)

### Geometric parameters (Å, º)

**Table:**

F1—C19	1.365 (2)	C8—C8A	1.453 (2)
O1—C1	1.2317 (18)	C8A—C9	1.514 (2)
O2—C8	1.2257 (18)	C9—C9A	1.508 (2)
C1—C9A	1.447 (2)	C9—C15	1.530 (2)
C1—C2	1.507 (2)	C9—H9	0.9800
C2—C3	1.531 (2)	N10—H10	0.8600
C2—H2A	0.9700	C11—H11A	0.9600
C2—H2B	0.9700	C11—H11B	0.9600
C3—C12	1.526 (2)	C11—H11C	0.9600
C3—C4	1.528 (2)	C12—H12A	0.9600
C3—C11	1.531 (2)	C12—H12B	0.9600
C4—C4A	1.498 (2)	C12—H12C	0.9600
C4—H4A	0.9700	C13—H13A	0.9600
C4—H4B	0.9700	C13—H13B	0.9600
C4A—C9A	1.358 (2)	C13—H13C	0.9600
C4A—N10	1.3766 (19)	C14—H14A	0.9600
C5—C5A	1.495 (2)	C14—H14B	0.9600
C5—C6	1.532 (2)	C14—H14C	0.9600
C5—H5A	0.9700	C15—C20	1.382 (2)
C5—H5B	0.9700	C15—C16	1.387 (2)
C5A—C8A	1.351 (2)	C16—C17	1.377 (3)
C5A—N10	1.3772 (19)	C16—H16	0.9300
C6—C7	1.525 (2)	C17—C18	1.369 (3)
C6—C13	1.529 (3)	C17—H17	0.9300
C6—C14	1.532 (2)	C18—C19	1.363 (3)
C7—C8	1.506 (2)	C18—H18	0.9300
C7—H7A	0.9700	C19—C20	1.367 (3)
C7—H7B	0.9700	C20—H20	0.9300
			
O1—C1—C9A	121.51 (14)	C8A—C9—C15	108.64 (12)
O1—C1—C2	120.49 (14)	C9A—C9—H9	108.7
C9A—C1—C2	117.97 (13)	C8A—C9—H9	108.7
C1—C2—C3	114.93 (13)	C15—C9—H9	108.7
C1—C2—H2A	108.5	C4A—C9A—C1	120.54 (14)
C3—C2—H2A	108.5	C4A—C9A—C9	120.86 (13)
C1—C2—H2B	108.5	C1—C9A—C9	118.53 (12)
C3—C2—H2B	108.5	C4A—N10—C5A	121.29 (12)
H2A—C2—H2B	107.5	C4A—N10—H10	119.4
C12—C3—C4	110.39 (14)	C5A—N10—H10	119.4
C12—C3—C11	109.23 (14)	C3—C11—H11A	109.5
C4—C3—C11	109.64 (14)	C3—C11—H11B	109.5
C12—C3—C2	110.11 (14)	H11A—C11—H11B	109.5
C4—C3—C2	108.35 (12)	C3—C11—H11C	109.5
C11—C3—C2	109.09 (13)	H11A—C11—H11C	109.5
C4A—C4—C3	112.70 (12)	H11B—C11—H11C	109.5
C4A—C4—H4A	109.1	C3—C12—H12A	109.5
C3—C4—H4A	109.1	C3—C12—H12B	109.5
C4A—C4—H4B	109.1	H12A—C12—H12B	109.5
C3—C4—H4B	109.1	C3—C12—H12C	109.5
H4A—C4—H4B	107.8	H12A—C12—H12C	109.5
C9A—C4A—N10	120.11 (13)	H12B—C12—H12C	109.5
C9A—C4A—C4	123.13 (14)	C6—C13—H13A	109.5
N10—C4A—C4	116.75 (12)	C6—C13—H13B	109.5
C5A—C5—C6	112.79 (13)	H13A—C13—H13B	109.5
C5A—C5—H5A	109.0	C6—C13—H13C	109.5
C6—C5—H5A	109.0	H13A—C13—H13C	109.5
C5A—C5—H5B	109.0	H13B—C13—H13C	109.5
C6—C5—H5B	109.0	C6—C14—H14A	109.5
H5A—C5—H5B	107.8	C6—C14—H14B	109.5
C8A—C5A—N10	120.17 (14)	H14A—C14—H14B	109.5
C8A—C5A—C5	123.37 (14)	C6—C14—H14C	109.5
N10—C5A—C5	116.45 (12)	H14A—C14—H14C	109.5
C7—C6—C13	110.98 (14)	H14B—C14—H14C	109.5
C7—C6—C14	109.44 (15)	C20—C15—C16	117.63 (16)
C13—C6—C14	109.17 (16)	C20—C15—C9	121.66 (13)
C7—C6—C5	107.34 (14)	C16—C15—C9	120.53 (15)
C13—C6—C5	110.50 (14)	C17—C16—C15	121.07 (19)
C14—C6—C5	109.37 (14)	C17—C16—H16	119.5
C8—C7—C6	114.23 (14)	C15—C16—H16	119.5
C8—C7—H7A	108.7	C18—C17—C16	121.17 (18)
C6—C7—H7A	108.7	C18—C17—H17	119.4
C8—C7—H7B	108.7	C16—C17—H17	119.4
C6—C7—H7B	108.7	C19—C18—C17	117.04 (18)
H7A—C7—H7B	107.6	C19—C18—H18	121.5
O2—C8—C8A	121.84 (15)	C17—C18—H18	121.5
O2—C8—C7	120.85 (15)	C18—C19—F1	118.19 (17)
C8A—C8—C7	117.28 (13)	C18—C19—C20	123.36 (19)
C5A—C8A—C8	120.10 (14)	F1—C19—C20	118.45 (17)
C5A—C8A—C9	120.50 (14)	C19—C20—C15	119.71 (16)
C8—C8A—C9	119.38 (12)	C19—C20—H20	120.1
C9A—C9—C8A	109.52 (12)	C15—C20—H20	120.1
C9A—C9—C15	112.41 (13)		
			
O1—C1—C2—C3	153.20 (15)	C8—C8A—C9—C15	−83.98 (16)
C9A—C1—C2—C3	−28.8 (2)	N10—C4A—C9A—C1	177.60 (14)
C1—C2—C3—C12	−69.81 (17)	C4—C4A—C9A—C1	−3.2 (2)
C1—C2—C3—C4	51.02 (18)	N10—C4A—C9A—C9	−5.5 (2)
C1—C2—C3—C11	170.33 (14)	C4—C4A—C9A—C9	173.74 (15)
C12—C3—C4—C4A	71.15 (17)	O1—C1—C9A—C4A	−178.70 (15)
C11—C3—C4—C4A	−168.46 (14)	C2—C1—C9A—C4A	3.3 (2)
C2—C3—C4—C4A	−49.50 (18)	O1—C1—C9A—C9	4.3 (2)
C3—C4—C4A—C9A	27.9 (2)	C2—C1—C9A—C9	−173.69 (14)
C3—C4—C4A—N10	−152.80 (14)	C8A—C9—C9A—C4A	25.2 (2)
C6—C5—C5A—C8A	19.5 (2)	C15—C9—C9A—C4A	−95.64 (17)
C6—C5—C5A—N10	−161.31 (14)	C8A—C9—C9A—C1	−157.80 (13)
C5A—C5—C6—C7	−49.58 (19)	C15—C9—C9A—C1	81.32 (17)
C5A—C5—C6—C13	71.57 (18)	C9A—C4A—N10—C5A	−14.6 (2)
C5A—C5—C6—C14	−168.23 (15)	C4—C4A—N10—C5A	166.08 (14)
C13—C6—C7—C8	−66.14 (19)	C8A—C5A—N10—C4A	11.1 (2)
C14—C6—C7—C8	173.31 (15)	C5—C5A—N10—C4A	−168.07 (14)
C5—C6—C7—C8	54.70 (19)	C9A—C9—C15—C20	33.0 (2)
C6—C7—C8—O2	153.47 (15)	C8A—C9—C15—C20	−88.35 (18)
C6—C7—C8—C8A	−28.6 (2)	C9A—C9—C15—C16	−152.02 (16)
N10—C5A—C8A—C8	−169.29 (14)	C8A—C9—C15—C16	86.61 (18)
C5—C5A—C8A—C8	9.8 (2)	C20—C15—C16—C17	0.7 (3)
N10—C5A—C8A—C9	12.3 (2)	C9—C15—C16—C17	−174.41 (18)
C5—C5A—C8A—C9	−168.58 (14)	C15—C16—C17—C18	0.0 (3)
O2—C8—C8A—C5A	172.64 (15)	C16—C17—C18—C19	−0.4 (3)
C7—C8—C8A—C5A	−5.3 (2)	C17—C18—C19—F1	179.82 (19)
O2—C8—C8A—C9	−8.9 (2)	C17—C18—C19—C20	0.2 (3)
C7—C8—C8A—C9	173.17 (14)	C18—C19—C20—C15	0.5 (3)
C5A—C8A—C9—C9A	−28.68 (19)	F1—C19—C20—C15	−179.10 (16)
C8—C8A—C9—C9A	152.89 (13)	C16—C15—C20—C19	−1.0 (3)
C5A—C8A—C9—C15	94.45 (16)	C9—C15—C20—C19	174.13 (16)

### Hydrogen-bond geometry (Å, º)

**Table:**

*D*—H···*A*	*D*—H	H···*A*	*D*···*A*	*D*—H···*A*
N10—H10···O1^i^	0.86	2.14	2.990 (2)	168

Symmetry code: (i) *x*, −*y*+1/2, *z*−1/2.
